# Predicting the reward value of faces and bodies from social perception

**DOI:** 10.1371/journal.pone.0185093

**Published:** 2017-09-19

**Authors:** Danielle Morrison, Hongyi Wang, Amanda C. Hahn, Benedict C. Jones, Lisa M. DeBruine

**Affiliations:** 1 Institute of Neuroscience and Psychology, University of Glasgow, Glasgow, United Kingdom; 2 Psychology Department, Humboldt State University, Arcata, California, United States of America; University of Bologna, ITALY

## Abstract

Social judgments of faces are thought to be underpinned by two perceptual components: valence and dominance. Recent work using a standard key-press task to assess reward value found that these valence and dominance components were both positively related to the reward value of faces. Although bodies play an important role in human social interaction, the perceptual dimensions that underpin social judgments of bodies and their relationship to the reward value of bodies are not yet known. The current study investigated these issues. We replicated previous studies showing that valence and dominance underpin social judgments of faces and that both components are positively related to the reward value of faces. By contrast, social judgments of bodies were underpinned by a single component that reflected aspects of both perceived valence and perceived dominance and was positively correlated with the reward value of bodies. These results highlight differences in how observers process faces and bodies.

## Introduction

Principal Component Analyses (PCA) of a wide range of trait ratings demonstrate that social judgments of faces are underpinned by two perceptual components: valence and dominance [1,2]. The valence component is highly correlated with traits such as trustworthiness and attractiveness and is thought to represent perceptions relating to intent to cause harm [1,2]. The dominance component is highly correlated with traits such as dominance and aggressiveness and is thought to represent perceptions relating to capacity to cause harm [1,2]. The role of these perceptual dimensions in social perception is not limited to social judgments of faces; social judgments of voices are also underpinned by orthogonal valence and dominance components [3].

Behavioral and neurobiological evidence shows that viewing faces high on traits that are positively correlated with the valence component, such as attractiveness and trustworthiness, is rewarding [4]. Using a standard key-press task that has been widely used to measure stimulus reward value [4], Wang et al. [2] recently showed that faces that scored higher on the valence component had greater reward value (i.e., participants chose to view these faces longer). Independent of the effect of valence, Wang et al. [2] also found that faces that scored higher on the dominance component had greater reward value. That the dominance component was positively related to the reward value of faces is consistent with research demonstrating that macaques find viewing the faces of dominant conspecifics rewarding [5].

The studies described above focused on the perceptual dimensions underpinning social judgments of static, neutral faces [1,2] and how those dimensions relate to reward value [2]. Although body characteristics also play an important role in social interaction and person perception [6], the perceptual dimensions underpinning social judgments of bodies and their relationships to reward value are not known. However, bodies may be rewarding because of the roles they play in mate preferences and intrasexual competitiveness. For example, men and women rated front-view and profile images of women similarly for attractiveness [7], suggesting that bodies provide information regarding sexual attraction between the sexes and the potential for comparison within sex regarding one’s own attractiveness. Since physical strength represents another aspect of intrasexual competitiveness and can be assessed via bodies [8], bodies possessing a particular level of strength may be rewarding to view.

Given the relationships between body perception, mate preferences, and intrasexual competitiveness, further research regarding the specific social perception of bodies and reward value is necessary. Consequently, the current study used PCAs of trait ratings of static, neutral bodies and a standard key-press task to investigate these issues. We also sought to replicate previous research reporting that social judgments of faces are underpinned by valence and dominance components [1,2] and that both components are positively correlated with the reward value of faces [2].

## Method

### Stimuli

Stimuli were face and nude body images of 50 white men (mean age = 24.12 years, SD = 3.03 years, range = 19 to 30 years; mean height = 181.42 cm, SD = 6.70, range = 168.00 to 200.00 cm; mean weight = 76.72 kg, SD = 10.44, range = 53.00 to 100.00 kg) and 50 white women (mean age = 24.1 years, SD = 3.01 years, range = 19 to 30 years; mean height = 168.98 cm, SD = 6.47, range = 155.00 to 184.00 cm; mean weight = 54.94 kg, SD = 6.26, range = 42.00 to 75.00 kg). All images were taken from the 3d.sk image set.

Face images were taken against neutral backgrounds. Individuals faced the camera with direct gaze and neutral expressions. Face images were aligned on pupil position and masked so only the face and ears were visible. Similar stimuli have been used in previous research on social perception of faces (e.g., Wang et al., 2016).

Body images were also taken against neutral backgrounds. Individuals faced the camera with their legs shoulder width apart and arms at 45-degree angles. Bodies were standardized relative to actual height. Faces and genitals were obscured. Similar stimuli have been used in previous research on social perception of bodies [9,10].

### Trait ratings

These images were rated by 449 men (mean age = 30.3 years, SD = 3.03 years) and 509 women (mean age = 26.1 years, SD = 3.01 years) who were recruited online between October 2015 and November 2016. Raters provided written consent and the researchers did not have access to any identifying rater information. These raters were randomly assigned to rate the male faces, male bodies, female faces, or female bodies for one of the 13 traits previously investigated by Oosterhof and Todorov [1] and Wang et al. [2]. These traits were aggressive, attractive, caring, confident, dominant, emotionally stable, intelligent, mean, responsible, sociable, trustworthy, happy, and weird. Following previous research on the dimensions underlying social judgments [2,3], this part of the study was run online, with participants recruited from links on social bookmarking websites (e.g., stumbleupon.com).

The procedure for ratings of the 13 traits was based on that used by Oosterhof and Todorov [1] and Wang et al. [2]. Participants were asked to "Please rate how [trait] this [face/body] is on a scale from 1 (much less [trait] than average) to 7 (much more [trait] than average)." Trial order was fully randomized. Each stimulus type (male faces, male bodies, female faces, female bodies) was rated for each trait by between 10 and 14 women and between 10 and 14 men. Although the majority (92.2%) of participants rated only one trait for one stimulus type, 7.8% of participants provided ratings for more than one combination of trait and stimulus type. Methods, stimuli, design, and analyses of the trait ratings task are pre-registered (https://osf.io/2j9qd/).

### Key-press task

Twenty-six men (mean age = 23.09 years, SD = 4.74 years) and 28 women (mean age = 26.45 years, SD = 10.07 years) were recruited via email from October to November 2016 and provided written consent. Researchers did not have access to identifying participant information. Participants completed a standard key-press task, similar to those used to assess image reward value in previous research [2,11,12]. Responses on this key-press task are a good predictor of neural measures of the reward value of social stimuli [12]. On this key-press task, participants could control the viewing duration of each stimulus by repeatedly pressing specified keyboard keys. Alternately pressing keys 7 and 8 increased the 4 second default viewing time (i.e., the length of time for which the image remained onscreen if no keys were pressed). Alternately pressing keys 1 and 2 decreased the default viewing time. Each key press increased or decreased the viewing time by 100ms. Participants completed a block of practice trials with unrelated stimuli before beginning the experimental trials. Stimuli were shown in four separate blocks of trials (male faces, male bodies, female faces, and female bodies). Block order and trial order within each block were fully randomized. This part of the study was run in the laboratory. Following previous studies using this key-press task [2,11,12], we calculated key-press scores for each trial by subtracting the number of key presses made to decrease viewing time from the number of key presses made to increase viewing time. Higher key-press scores indicate greater reward value [2,11,12]. While the stimuli were from the trait ratings task and the methodology followed Wang et al. [2], the analyses were not pre-registered.

## Results

### Are the perceptual dimensions underpinning social judgments of faces and bodies identical?

To investigate this issue, we first calculated the mean rating for every male face, male body, female face, and female body separately for each trait. All data and analysis scripts are also publically available (https://osf.io/g27wf/). Estimates of inter-rater agreement for each combination of trait and stimulus type, calculated using Cronbach’s alpha, are given in Table 1. Inter-rater agreement for all face ratings was high (all Cronbach’s alphas > 0.71). Inter-rater agreement for all body ratings was generally high (i.e., Cronbach’s alphas > .70), with the exception of aggressiveness, intelligence, meanness, and trustworthiness ratings of female bodies and trustworthiness and weirdness ratings of male bodies (all Cronbach’s alphas for these traits < .70). These traits with low reliability were excluded from all subsequent analyses.

**Table 1:** Inter-rater agreement (Cronbach's alpha) for trait ratings of faces and bodies.

Trait	Male Face	Male Body	Female Face	Female Body
aggressive	0.86	0.87	0.76	0.63
attractive	0.82	0.91	0.84	0.88
caring	0.89	0.81	0.90	0.70
confident	0.84	0.94	0.82	0.87
dominant	0.82	0.93	0.72	0.80
emotionally stable	0.85	0.74	0.82	0.73
happy	0.94	0.85	0.93	0.81
intelligent	0.78	0.72	0.71	0.56
mean	0.82	0.78	0.78	0.53
responsible	0.80	0.78	0.79	0.70
sociable	0.81	0.87	0.82	0.85
trustworthy	0.82	0.35	0.80	0.60
weird	0.91	0.48	0.87	0.77

Following Oosterhof and Todorov [1] and Wang et al. [2], we then analyzed the mean trait ratings using Principal Component Analyses (PCA). Separate PCAs, reporting components with eigenvalues above one, were conducted for male faces, male bodies, female faces, and female bodies. Tables 2 and 3 show the correlations between each component and each of the individual traits for male and female stimuli separately.

**Table 2:** Correlations between each component and individual traits for male stimuli.

Trait	Face Valence Component	Face Dominance Component	Body General Component
aggressive	-0.578	0.741	0.850
attractive	0.771	0.413	0.948
caring	0.889	-0.307	0.860
confident	0.678	0.507	0.973
dominant	0.086	0.867	0.898
emotionally stable	0.901	-0.061	0.931
happy	0.772	-0.243	0.896
intelligent	0.705	0.154	0.882
mean	-0.580	0.745	0.848
responsible	0.734	0.170	0.893
sociable	0.837	0.138	0.952
trustworthy	0.875	-0.072	NA
weird	-0.676	-0.523	NA

**Table 3:** Correlations between each component and individual traits for female stimuli.

Trait	Face Valence Component	Face Dominance Component	Face Geekiness Component	Body General Component
aggressive	-0.636	0.655	0.004	NA
attractive	0.724	0.438	-0.403	0.938
caring	0.852	-0.336	0.035	0.856
confident	0.590	0.642	0.273	0.938
dominant	-0.235	0.860	0.186	0.902
emotionally stable	0.763	0.476	0.082	0.868
happy	0.874	0.088	0.145	0.915
intelligent	0.668	0.121	0.464	NA
mean	-0.566	0.751	-0.005	NA
responsible	0.791	-0.019	0.351	0.800
sociable	0.780	0.318	-0.380	0.940
trustworthy	0.848	-0.339	-0.031	NA
weird	-0.614	-0.273	0.503	-0.877

#### Male stimuli

The PCA of trait ratings for male faces produced two components. The first component explained 53% of the variance in ratings and was highly correlated with the traits trustworthiness, caringness, and emotional stability (see Table 2). The second component explained 21% of the variance in ratings and was highly correlated with the traits dominance, meanness, and aggressiveness (see Table 2). By contrast with these results for male faces, PCA of trait ratings for male bodies produced only one component. This component explained 82% of the variance in ratings and was highly correlated with all traits analyzed (see Table 2). Following Oosterhof and Todorov [1] and Wang et al. [2], we labeled the two face components *male valence* and *male dominance*. We labeled the single body component, the *male body general component*.

#### Female stimuli

The PCA of trait ratings for female faces produced three components. The first component explained 50% of the variance in ratings and was highly correlated with the traits happiness, caringness, and trustworthiness (see Table 3). The second component explained 23% of the variance in ratings and was highly correlated with the traits dominance, meanness, and aggressiveness (see Table 3). The third component explained 8% of the variance in ratings and was correlated with the traits weirdness and intelligence, albeit relatively weakly (see Table 3). By contrast with these results for female faces, PCA of trait ratings for female bodies produced only one component. This component explained 80% of the variance in ratings and was highly correlated with all traits analyzed (see Table 3). Following Oosterhof and Todorov [1] and Wang et al. [2], we labeled the first two face components *female valence* and *female dominance*. Because it was correlated with weirdness and intelligence, we labeled the third face component *female geekiness*. We labeled the single female body component, the *female body general component*.

Thus, PCAs of trait ratings of men’s and women’s faces and bodies suggest that different dimensions underpin social judgments of faces and bodies. While social judgments of faces were largely underpinned by orthogonal valence and dominance components, social judgments of bodies were underpinned by a single component that was highly correlated with traits relevant to both valence and dominance.

For male stimuli, the body general component was not correlated with the face valence component (r = 0.183, p = 0.203) or the face dominance component (r = 0.206, p = 0.152). For female stimuli, the body general component was not correlated with the face valence component (r = -0.033, p = 0.818), the face dominance component (r = 0.014, p = 0.925), or the face geekiness component (r = 0.083, p = 0.566).

### How are the perceptual dimensions underpinning social judgments of faces and bodies related to their reward value?

We investigated this issue using linear mixed models. Analyses were conducted in the programming software R, version 3.4.0 in conjunction with lme4 and lmerTest [13,14,15]. Because the perceptual dimensions were not comparable, separate models were conducted for the reward value of male faces, male bodies, female faces, and female bodies. The dependent variable in each model was key-press score, which was right-skewed. Following recommendations by Emerson [16] and Emerson and Soto [17], we therefore log transformed key-press scores after adding an optimal constant to make all values positive and scaling the original scores. Each model included participant sex (effect coded so that male = -0.5, female = +0.5), the principal components from the analyses described above for the relevant stimulus type, and all possible interactions among these variables. Following recommendations from Barr, Levy, Scheepers, and Tily [18], maximal models were specified. Random intercepts were specified for each stimulus and participant. Random slopes by stimulus were specified for participant sex. Random slopes by participant were specified for each social perception component and all of their interactions. The full specifications and results for each model are given in our Supplemental Materials.

#### Male faces

**Fig 1:**
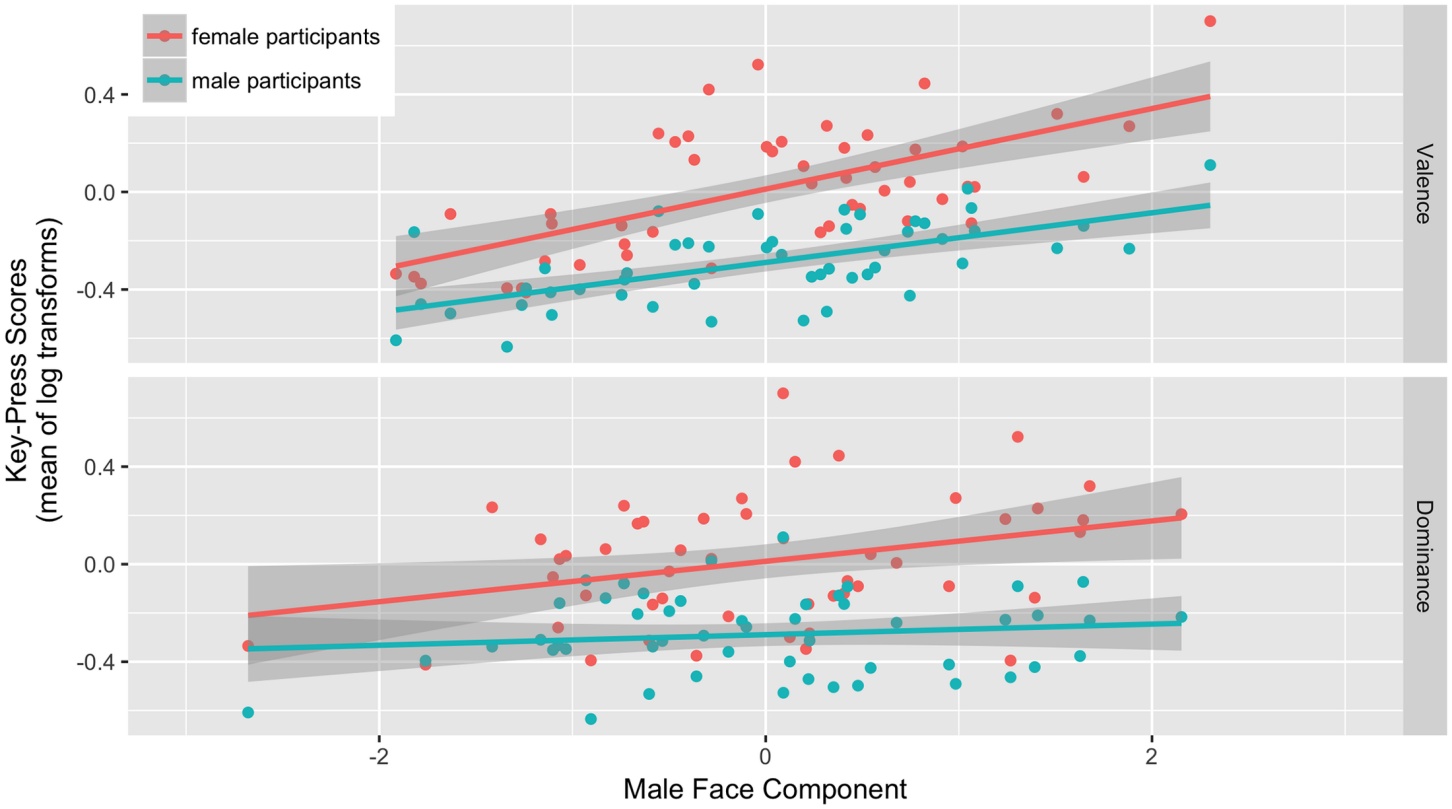
The effects of the valence and dominance components on key-press scores for male faces.

The model for male faces showed significant positive effects of the male valence component (beta = 0.135, CI [0.085, 0.184], p < .001) and the male dominance component (beta = 0.054, CI [0.013, 0.096], p = 0.012). The interaction between participant sex and the male dominance component was also significant (beta = 0.073, CI [0.005, 0.142], p = 0.037). The interaction between participant sex and the male dominance component reflected the positive effect of male dominance on key-press scores being stronger for female than male participants (see Fig 1). There were no other significant effects (all ps ≥ 0.114).

#### Male bodies

**Fig 2:**
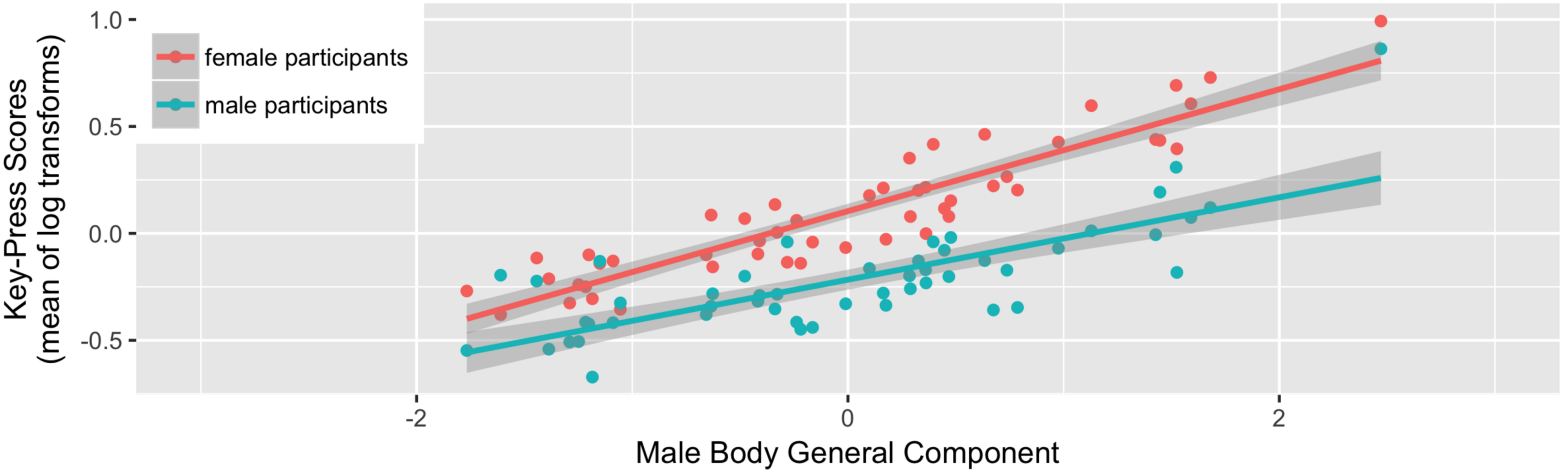
The significant main effect of the body general component on key-press scores for male bodies.

The model for male bodies showed a significant positive effect of the male body general component (beta = 0.239, CI [0.174, 0.303], p < .001). There were no other significant effects (both ps ≥ 0.062). The relationship between key-press scores and the male body general component is shown in Fig 2.

#### Female faces

**Fig 3:**
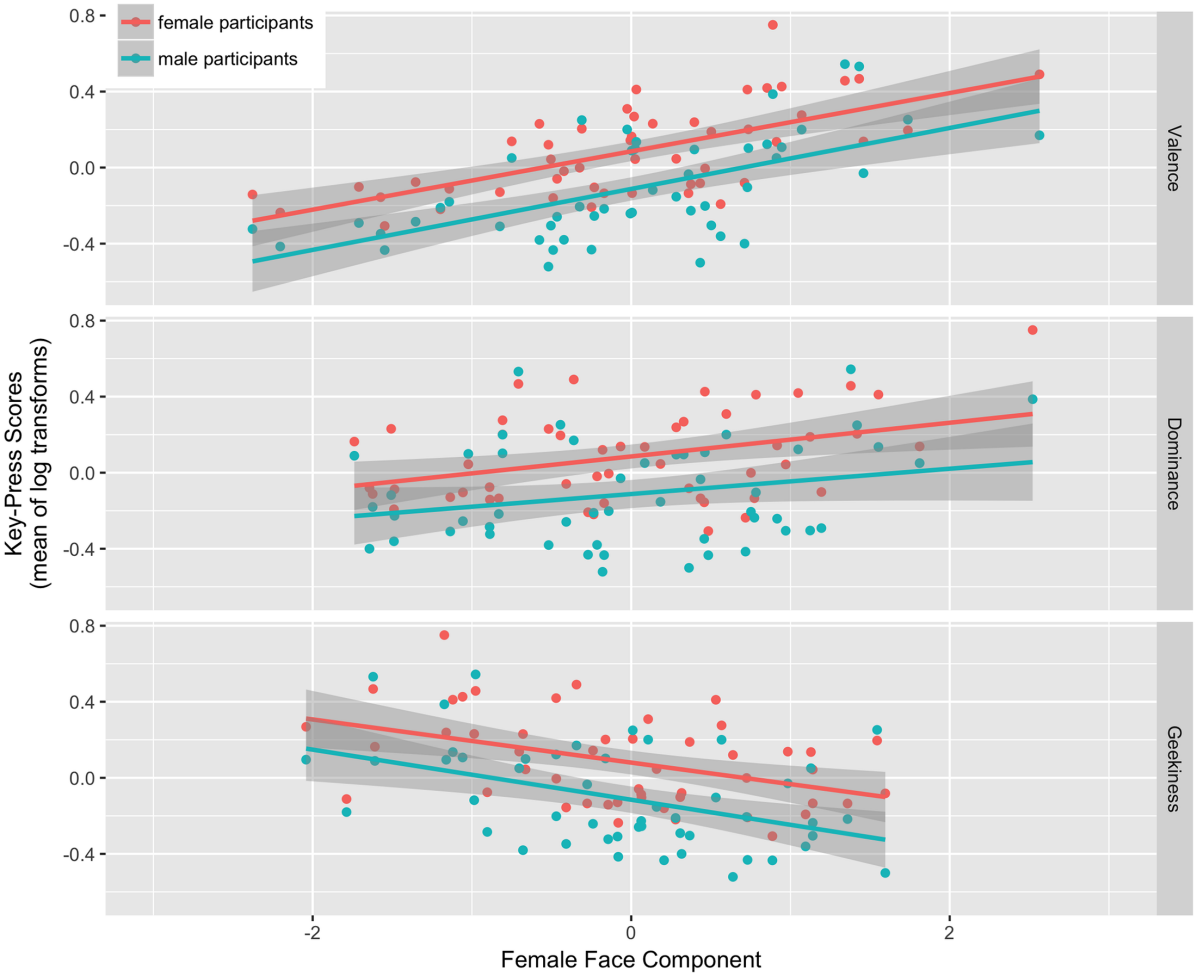
The main effects of the valence, dominance, and geekiness components on key-press scores for female faces.

The model for female faces showed a significant positive effect of the female valence component (beta = 0.156, CI [0.114, 0.197], p < .001), a significant positive effect of the female dominance component (beta = 0.071, CI [0.026, 0.117], p = 0.003), and a significant negative effect of the female geekiness component (beta = -0.104, CI [-0.146, -0.061], p < .001). There were no other significant effects (all ps ≥ 0.188). The relationships between key-press scores and the female valence, female dominance, and female geekiness components are shown in Fig 3.

#### Female bodies

**Fig 4:**
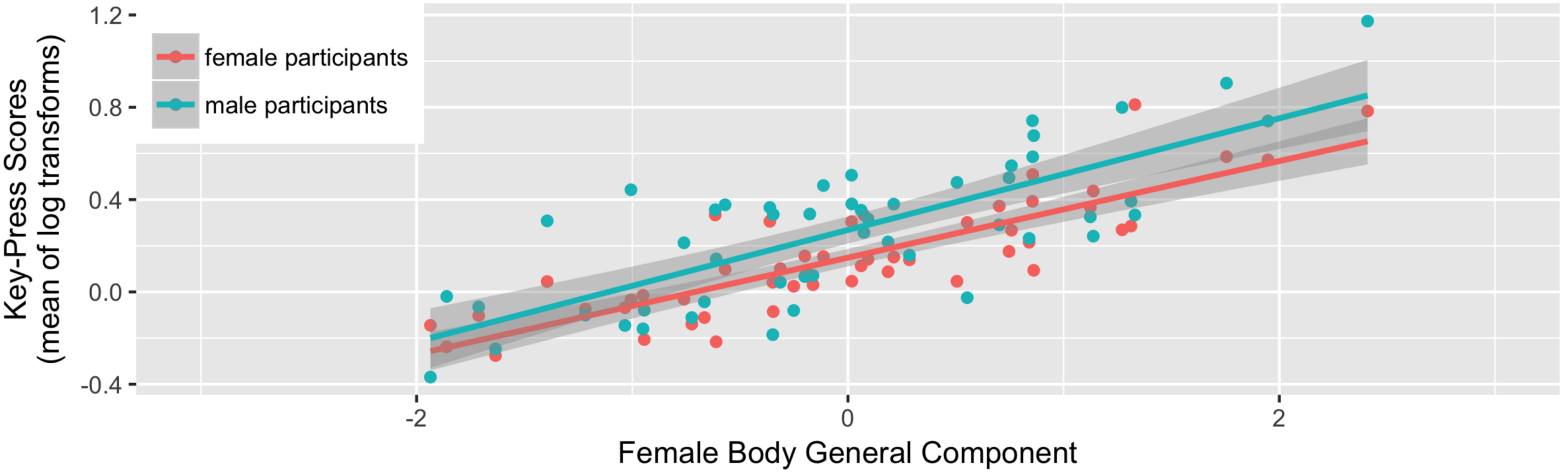
The main effect of the body general component on key-press scores for female bodies.

The model for female bodies showed a significant positive effect of the female body general component (beta = 0.226, CI [0.160, 0.291], p < .001). There were no other significant effects (both ps ≥ 0.427). The relationship between key-press scores and the female body general component is shown in Fig 4.

Key-press scores for faces and bodies were positively correlated for male stimuli (r = 0.298, p = 0.035), but not female stimuli (r = -0.120, p = 0.407).

## Discussion

Our Principal Component Analyses (PCAs) of trait ratings of both male and female faces showed that much of the variance in social judgments of faces (74% in male faces and 73% in female faces) was explained by two components: one that is highly correlated with traits such as trustworthiness and caringness (the valence component) and the other that is highly correlated with traits such as dominance and aggressiveness (the dominance component). These results are very similar to those reported by Oosterhof and Todorov [1] and Wang et al. [2], who also found that social judgments of faces were underpinned by valence and dominance components. Consistent with previous research suggesting that viewing faces possessing traits highly correlated with the valence component is rewarding, analyses of participants’ responses on a standard key-press task also showed that the valence component was positively correlated with the reward value of faces. Replicating Wang et al. [2], we also found that the dominance component was independently related to the reward value of faces. Thus, our analyses present further evidence that the reward value of faces is contingent on the independent contributions of at least two perceptual dimensions (valence and dominance). Our PCA of trait ratings of female faces revealed a third component (correlated with intelligence and weirdness, labelled the geekiness component). This third component was also negatively related to the reward value of female faces, presenting converging evidence that the reward value of faces is not solely determined by their perceived valence.

By contrast with the results of our PCAs of trait ratings of face stimuli, much of the variance in social judgments of bodies (82% in male bodies and 80% in female bodies) was explained by a single, general component that was highly correlated with attractiveness, dominance, and caringness. For both male and female stimuli, this body component was positively correlated with key-press scores. These results suggest that, by contrast with the reward value of static, neutral faces, the single general perceptual dimension, derived from trait ratings used here and in previous studies, predicts the reward value of static, neutral bodies. Thus, while there may be similarities in many aspects of observers’ responses to face and body stimuli [19,20], our results show that the perceptual dimensions related to the reward value of faces and bodies are qualitatively different.

It is important to note that certain traits (i.e., male bodies: trustworthy and weird; female bodies: aggressive, intelligent, mean, and trustworthy) were not included in body analyses because of their low inter-rater agreement, suggesting that these bodies may not provide information relevant to these trait judgements. Therefore, body perception may not be definable by the same intent and ability to cause harm (i.e., valence and dominance) as face perception. The present analyses used established trait ratings relevant specifically to faces in order to determine whether the social perception of bodies followed the same pattern as faces and voices. Previous work has also used similar traits to determine that social perception of voices can be encapsulated by valence and dominance [3]. In order to examine body perception specifically, and not just whether it differs from face perception, future research could use a bottom-up approach starting with free description of bodies that could then be grouped into relevant traits, as was the procedure in Oosterhof and Todorov [1]. The trait ratings based on this new list of traits specific to body perception could allow for a more nuanced understanding of body perception. Just as previous research on faces focuses on unadorned, static faces with neutral expressions, here we also focused on unadorned, static bodies with neutral poses. Including other variables, such as body pose, may also highlight other aspects of body perception or may provide information regarding intent and ability to cause harm not present in static, neutral bodies.

Previous research reported that attractiveness ratings of nude bodies and faces were positively correlated [9,10]. That is, men and women who possessed attractive faces also tended to possess attractive bodies. These results have been interpreted as evidence that bodies and faces convey common information, potentially about an individual's health [9,10]. By contrast with this finding, the body component was not correlated with any of the face components in male or female stimuli.

In summary, we find that the reward value of faces is positively and independently correlated with perceptions of facial valence and dominance. By contrast, the reward value of bodies is positively correlated with a single, general perceptual component that reflects aspects of both the perceived valence and perceived dominance of bodies. These results highlight differences in how observers process faces and bodies.

## Supporting information

S1 Table Variable names legend. (DOCX)

S2 Table Full results of model testing for effects of male valence and dominance components on key-press scores for male faces. (DOCX)

S3 Table Full results of model testing for effects of male body general component on key-press scores for male bodies. (DOCX)

S4 Table Full results of model testing for effects of female valence and dominance components on key-press scores for female faces. (DOCX)

S5 Table Full results of model testing for effects of female body general component on key-press scores for female bodies. (DOCX)
