# Supplementary material for: Predicting the reward value of faces and bodies from social perception
Source: PLoS One. 2017 Sep 19;12(9):e0185093. doi: 10.1371/journal.pone.0185093 (PMC5604994; doi:10.1371/journal.pone.0185093)
Supplement: S1 Table — (DOCX) [file pone.0185093.s001.docx]

**S1 Table. Variable names legend.**

| Table Component Names | Actual Component Names |
| --- | --- |
| PCval | face valence component |
| PCdom | face dominance component |
| PCgeek | face geekiness component |
| PCbody | body general component |
