# Supplementary material for: Predicting the reward value of faces and bodies from social perception
Source: PLoS One. 2017 Sep 19;12(9):e0185093. doi: 10.1371/journal.pone.0185093 (PMC5604994; doi:10.1371/journal.pone.0185093)
Supplement: S2 Table — (DOCX) [file pone.0185093.s002.docx]

**S2 Table. Full results of model testing for effects of male valence and dominance components on key-press scores for male faces.**

|  | Estimate | Standard Error | Degrees of Freedom | t value | p value |
| --- | --- | --- | --- | --- | --- |
| PCval | 0.135 | 0.025 | 67.784 | 5.409 | < .001 |
| PCdom | 0.054 | 0.021 | 54.475 | 2.611 | 0.012 |
| Participant Sex | 0.301 | 0.189 | 56.459 | 1.596 | 0.116 |
| PCval x PCdom | 0.006 | 0.018 | 48.767 | 0.336 | 0.738 |
| PCval x Participant Sex | 0.071 | 0.044 | 55.974 | 1.608 | 0.114 |
| PCdom x Participant Sex | 0.073 | 0.034 | 46.052 | 2.142 | 0.037 |
| PCval x PCdom x Participant Sex | 0.038 | 0.027 | 48.541 | 1.372 | 0.176 |
