# Supplementary material for: Predicting the reward value of faces and bodies from social perception
Source: PLoS One. 2017 Sep 19;12(9):e0185093. doi: 10.1371/journal.pone.0185093 (PMC5604994; doi:10.1371/journal.pone.0185093)
Supplement: S3 Table — (DOCX) [file pone.0185093.s003.docx]

**S3 Table. Full results of model testing for effects of male body general component on key-press scores for male bodies.**

|  | Estimate | Standard Error | Degrees of Freedom | t value | p value |
| --- | --- | --- | --- | --- | --- |
| PCbody | 0.239 | 0.033 | 68.287 | 7.332 | < .001 |
| Participant Sex | 0.320 | 0.168 | 56.000 | 1.906 | 0.062 |
| PCbody x Participant Sex | 0.092 | 0.060 | 55.977 | 1.536 | 0.130 |
