# Supplementary material for: Predicting the reward value of faces and bodies from social perception
Source: PLoS One. 2017 Sep 19;12(9):e0185093. doi: 10.1371/journal.pone.0185093 (PMC5604994; doi:10.1371/journal.pone.0185093)
Supplement: S4 Table — (DOCX) [file pone.0185093.s004.docx]

**S4 Table. Full results of model testing for effects of female valence and dominance components on key-press scores for female faces.**

|  | Estimate | Standard Error | Degrees of Freedom | t value | p value |
| --- | --- | --- | --- | --- | --- |
| PCval | 0.156 | 0.021 | 55.349 | 7.428 | < .001 |
| PCdom | 0.071 | 0.023 | 58.649 | 3.124 | 0.003 |
| PCgeek | -0.104 | 0.021 | 55.727 | -4.857 | < .001 |
| Participant Sex | 0.204 | 0.213 | 56.164 | 0.961 | 0.341 |
| PCval x PCdom | 0.015 | 0.023 | 48.645 | 0.636 | 0.527 |
| PCval x PCgeek | -0.018 | 0.025 | 49.289 | -0.710 | 0.481 |
| PCdom x PCgeek | -0.004 | 0.020 | 48.645 | -0.205 | 0.838 |
| PCval x Participant Sex | -0.006 | 0.035 | 60.154 | -0.167 | 0.868 |
| PCdom x Participant Sex | 0.023 | 0.038 | 74.935 | 0.598 | 0.552 |
| PCgeek x Participant Sex | 0.032 | 0.036 | 63.740 | 0.902 | 0.370 |
| PCval x PCdom x PCgeek | -0.009 | 0.028 | 44.498 | -0.314 | 0.755 |
| PCval x PCdom x Participant Sex | 0.046 | 0.035 | 2426.459 | 1.316 | 0.188 |
| PCval x PCgeek x Participant Sex | -0.005 | 0.039 | 69.793 | -0.135 | 0.893 |
| PCdom x PCgeek x Participant Sex | 0.006 | 0.031 | 2426.459 | 0.189 | 0.850 |
| PCval x PCdom x PCgeek x Participant Sex | 0.033 | 0.042 | 114.190 | 0.785 | 0.434 |
