# Supplementary material for: Predicting the reward value of faces and bodies from social perception
Source: PLoS One. 2017 Sep 19;12(9):e0185093. doi: 10.1371/journal.pone.0185093 (PMC5604994; doi:10.1371/journal.pone.0185093)
Supplement: S5 Table — (DOCX) [file pone.0185093.s005.docx]

**S5 Table. Full results of model testing for effects of female body general component on key-press scores for female bodies.**

|  | Estimate | Standard Error | Degrees of Freedom | t value | p value |
| --- | --- | --- | --- | --- | --- |
| PCbody | 0.226 | 0.033 | 65.926 | 6.807 | < .001 |
| Participant Sex | -0.120 | 0.150 | 57.266 | -0.799 | 0.427 |
| PCbody x Participant Sex | -0.033 | 0.063 | 59.269 | -0.514 | 0.609 |
